# Phospholipid binding residues of eukaryotic membrane-remodelling F-BAR domain proteins are conserved in *Helicobacter pylori* CagA

**DOI:** 10.1186/1756-0500-7-525

**Published:** 2014-08-13

**Authors:** Anna Roujeinikova

**Affiliations:** Department of Microbiology, Monash University, Building 76, Monash University, Clayton, Victoria 3800 Australia; Department of Biochemistry and Molecular Biology, Monash University, Building 76, Monash University, Clayton, Victoria 3800 Australia

**Keywords:** Cytotoxin-associated gene product A, Lipid binding, Membrane tethering, Lipid specificity, Functional homology

## Abstract

**Background:**

Cytotoxin associated gene product A (CagA) is an oncogenic protein secreted by the gastric bacterium *Helicobacter pylori*. Internalization of CagA by human epithelial cells occurs by an unknown mechanism that requires interaction with the host membrane lipid phosphatidylserine.

**Findings:**

Local homology at the level of amino acid sequence and secondary structure has been identified between the membrane-tethering region of CagA and the lipid-binding Fes-CIP4 homology-Bin/Amphiphysin/Rvs (F-BAR) domains of eukaryotic proteins. The F-BAR proteins are major components of the endocytic machinery. In addition to the membrane-binding F-BAR domains, they contain other domains that interact with actin-regulatory networks and mediate interplay between membrane dynamics and cytoskeleton re-arrangements. Positively charged residues found on the lipid binding face of the F-BAR domains are conserved in CagA and represent residues involved in CagA binding to lipids.

**Conclusions:**

The homologies with F-BAR proteins extend to lipid binding specificities and involvement in reorganization of the actin cytoskeleton. CagA and F-BAR domains share binding specificity for phosphatidylserine and phosphoinositides. Similar to the F-BAR proteins, CagA has a membrane-binding module and a module that shares structural homology with actin-binding proteins, and, like eukaryotic F-BAR domain proteins, CagA function is linked to actin dynamics. The uncovered similarities between the bacterial effector protein and eukaryotic F-BAR proteins suggest convergent evolution of CagA towards a similar function.

**Electronic supplementary material:**

The online version of this article (doi:10.1186/1756-0500-7-525) contains supplementary material, which is available to authorized users.

## Findings

### Introduction

*Helicobacter pylori* is a Gram negative pathogenic bacterium that infects the stomach tissue of approximately half the world’s population [1] and is associated with different gastric diseases ranging from gastritis to peptic ulcers and adenocarcinoma cancer [2–4]. Gastric cancer is the second leading cause of cancer deaths worldwide. Cytotoxin-associated gene product A (CagA) is a major virulence factor of *H. pylori*. Pathogenic strains of *H. pylori*, associated with the development of adenocarcinoma in humans, inject CagA into gastric epithelial cells, where it interacts with many different host cell proteins (*e.g.* Abl kinases, SRC, PAR1b/MARK2 kinases, CrkII, SHP-2 protein tyrosine phosphatase), interfering with signalling pathways that regulate cell growth and motility [5–10]. The CagA-mediated sustained deregulation of these pathways eventually leads to apoptosis in gastric epithelial cells and cancer.

Although the cellular effects of CagA are well-characterized, the structure-function relationship of this protein remains poorly understood. The *cagA* gene belongs to a 40 kb genetic locus called the cytotoxin-associated gene pathogenicity island (cag-PAI), which is hypothesized to have been acquired by horizontal gene transfer from an unrelated species [11]. In addition to the *cagA* gene, cag-PAI contains genes that encode for the components of a type IV secretion system (T4SS) which is responsible for translocating CagA into the host gastric epithelial cells [12]. Previous studies by Murata-Kamiya and co-workers [13] showed that inhibition of actin polymerization impaired CagA delivery into human epithelial cells, indicating that CagA internalization is dependent on host cell machinery and involves actin polymerisation. However, the mechanism by which CagA traverses the host cell membrane remains to be elucidated.

Internalization of CagA by host epithelial cells requires its interaction with host membrane lipid phosphatidylserine (PS) [13] and results in localization of CagA to the PS-rich inner leaflet of the host cell membrane [13, 14]. Membrane tethering is absolutely required for all CagA activities reported to date [6, 14, 15]. Interestingly, PS is physiologically present only on the inner leaflet of eukaryotic cell membranes; however, it has been shown to transiently externalize to the outer leaflet of the host plasma membrane at the sites of direct contact with *H. pylori* [14] *.* It is known that CagA exploits PS at both the outer and inner leaflets for entry into the host cell and localization to the plasma membrane, especially in polarized epithelial cells [13].

Previous site-directed mutagenesis studies revealed that CagA residues R619 and R621 (strain NCTC11637 numbering) are essential for binding to PS, uptake of CagA by the host cells and its association with the host cell membrane [13]. Analysis of the crystal structure of CagA fragment 1–876 revealed that the corresponding residues in strain 26695 (R624 and R626) are located in one of the α-helices (α18) of Domain II and, together with lysine residues at positions 613, 614, 617, 621, 631, 635, 636 of the same α-helix, form a positively charged patch on the CagA surface [16]. Systematic site-directed mutagenesis studies revealed that these positively charged residues are involved in the CagA-PS interaction in addition to R624 and R626 (strain 26695 numbering) [16]. It has been hypothesized that the positively charged face of the α-helix 610–639 (α18) tethers CagA to the negatively charged phosphate groups of the lipid membrane *via* electrostatic interactions.

To begin to understand the molecular mechanisms underpinning the internalization of CagA by human epithelial cells, the sequence and structural characteristics of CagA were analysed in comparison to those of other proteins. Local homology at the level of amino acid sequence and secondary structure has been identified between an α-helical region of CagA and the membrane-targeting region of the Fes-CIP4 homology-Bin/Amphiphysin/Rvs (F-BAR) domain of human proteins. The analysis presented here reveals that the homologies with F-BAR proteins extend to lipid binding specificities and involvement in reorganization of the actin cytoskeleton, altogether suggesting convergent evolution of CagA to a similar function.

## Methods

Analysis of the amino acid sequence of CagA from *H. pylori* strain 26695 (UniprotKB P55980) using the NCBI Conserved Domain Architecture Retrieval Tool (CDART) (http://www.ncbi.nlm.nih.gov/Structure/lexington/lexington.cgi) [17] identified a local homology between CagA residues 613–641 and region 231–259 within the F-BAR domain of human GAS7 (UniProtKB GAS7_HUMAN) and the corresponding region in GAS7 homologs from chicken (NCBI XP_415577.2), zebrafish (NCBI XP_001333507.2), sea squirt (NCBI XP_002123389) and African clawed frog (NP_001090555.1). Analysis of the local sequence alignment of CagA and GAS7 over this region revealed that conserved positively charged residues (lysine, arginine) implicated in the binding of F-BAR domains to the membrane are also present (and conserved) in the CagA sequence. The multiple sequence alignment was then extended to include the sequences of other F-BAR proteins: proline-serine-threonine phosphatase-interacting protein 1 (PSTPIP1); human formin-binding protein 17 (FBP17); and FCH domain only proteins 1 and 2 (FCHo1, FCHo2)) using ClustalW2 (http://www.ebi.ac.uk/Tools/msa/clustalw2/). Sequence-based prediction of the secondary structure of GAS7 was performed using the Jpred3 server (http://www.compbio.dundee.ac.uk/www-jpred/) [18]. The homology model of the F-BAR domain of human PSTPIP1 was constructed using MODELLER (9v12) [19, 20] based on the coordinates of the 2.3-Å resolution crystal structure of human FCHo2 (RSCB PDB ID 2v0o) [21]. Structure figures were prepared using PYMOL [22].

## Results

### Local sequence homology and the role of the conserved positively charged residues in CagA and F-BAR domains

**Figure 1:**
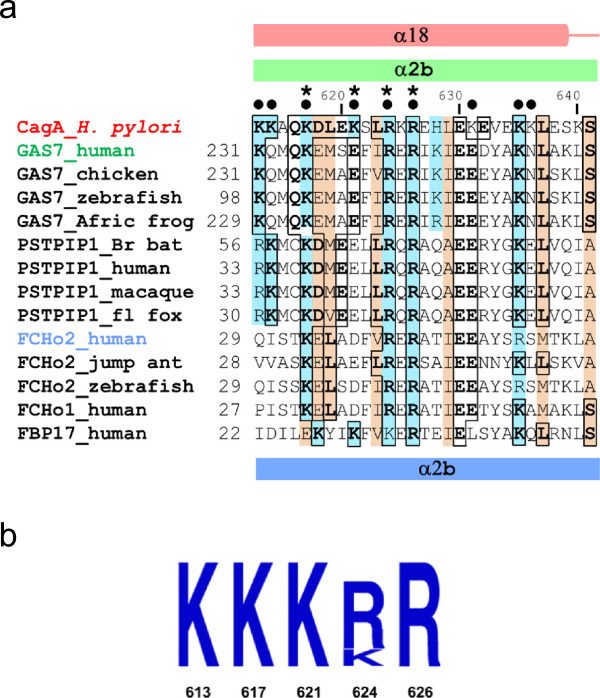
**Phospholipid binding residues of eukaryotic BAR domain proteins are conserved in** ***H. pylori*** **CagA. (a)** Local sequence alignment of CagA and F-BAR domains of representative eukaryotic proteins. The sequences are shown for: *H. pylori* CagA strain 26695 (UniprotKB P55980); growth arrest-specific protein 7 (GAS-7) from human (UniProtKB GAS7_HUMAN), chicken (NCBI XP_415577.2), zebrafish (NCBI XP_001333507.2), African clawed frog (NP_001090555.1); proline-serine-threonine phosphatase-interacting protein 1 (PSTPIP1) from Brandt's bat (UniProtKB S7PAL8_MYOBR), human (J3KPG6_HUMAN), rhesus macaque (UniProtKB H9ZF21_MACMU), black flying fox (UniProtKB L5JZ07_PTEAL); FCH domain only protein 2 (FCHo2) from human (NCBI NP_620137.2), Jerdon's jumping ant (Uniprot E2C5A9_HARSA), zebrafish (Uniprot F1RDR3_DANRE); FCH domain only protein 1 (FCHo1) from human (Uniprot FCHO1_HUMAN); and human formin-binding protein 17 (FBP17) (UniProtKB Q96RU3.2). Residue numbering above the sequences corresponds to CagA. Conserved residues are boxed and shown in bold. Positively charged conserved residues and their conservative substitutions are highlighted in blue. Other types of semi-conserved residues and their conservative substitutions are highlighted in orange. The positions of the amino acid substitutions associated with loss of PS-binding activity in CagA are shown by black dots. The positions of the substitutions associated with loss of lipid-binding activity in F-BAR domains are shown by stars. The predicted secondary structure of human GAS is shown in green above the sequences (cylinders represented α-helices). The secondary structure of CagA and FCHo2 derived from their respective crystal structures [16, 21] are shown in red and blue, and helices are labelled as in [16, 21]. **(b)** Conservation of the CagA positively charged residues equivalent to the membrane-binding residues of BAR domains. CagA amino acid sequences from 44 strains were aligned (Additional file 1) and the residue variability was plotted using a logo representation where the height of the stack indicates the sequence conservation at a given position, and the size of the letter denotes a residue’s relative frequency at that position among homologues.

A similarity search based on domain architecture implemented in CDART revealed that region 613–641 of the amino acid sequence of *H. pylori* CagA shares limited homology with the second α-helix (α2) of the F-BAR domain of the human protein GAS7. F-BAR domains are found in many eukaryotic proteins involved in membrane remodelling processes. They bind to the negatively charged surface of the lipid bilayer *via* an extensive positively charged patch on their surface [23–29]. Figure 1a shows local alignment of the sequences of CagA, and GAS7 and other representative members of the F-BAR domain subfamily, highlighting the residues implicated in lipid binding. CagA region 613–641, region 231–259 of human GAS7 and the respective regions in GAS7 from different eukaryotic species (chicken, zebrafish, sea squirt and African clawed frog) show significant (35%) sequence identity. Extending the analysis to include F-BAR domains of other proteins showed that, for example, CagA region 613–641 shares 38% and 31% sequence identity with the corresponding regions of PSTPIP1 and FCHo2, respectively. Alignment of the sequences of full-length CagA and F-BAR domains indicated that the detected homology is limited to this local region and does not extend beyond it. However, it was striking that many positively charged residues found on the lipid binding face of the F-BAR domains are conserved in CagA (K613, K614, K617, K621, R624, R626, K635) and represent residues involved in CagA binding to lipids (Figure 1a).

Previous genetic and structural studies on BAR and F-BAR domains have shown that their binding to phospholipid membranes is underpinned by electrostatic interactions involving conserved positively charged residues on their concave surface formed by helices α2 and α3 [21, 23, 30]. The identified region in F-BAR domains that shows homology with CagA forms part of helix α2. The replacement of K33 in this region in FBP17 with glutamate significantly reduced its membrane binding and tubulation activity *in vitro* and abolished the membrane invagination induced by GFP-FBP17 *in vivo* [23]. The K33Q + R35Q variant of this protein showed reduced phospholipid-binding ability and liposome tubulation [31]. Residues K37 and K44 (equivalent to K27 and K33 in FBP17) were shown to contribute to the membrane binding of the F-BAR domain of Syp1 [29]. Furthermore, the mouse syndapin residue R46 (equivalent to R35 in FBP17) was also implicated in membrane binding [24]. The lists of positively charged residues within this region that are implicated in membrane binding and deformation by the F-BAR domains of human FCHo2 and human FBP17 are given in Table 1.

**Table 1:** **F-BAR membrane-binding residues conserved between F-BAR and CagA**

FCHo2	FBP17	CagA	References
K33	K27	K617	26, 29 (K37 in Syp1)
Not conserved	K30	K621	26
R40	K33	R624	23, 26, 29, 31 (K44 in Syp1)
R42	R35	R626	31
Not conserved	K52	K644	31

Analysis of the multiple sequence alignment between CagA region 613–641 and the respective region in the F-BAR domains of GAS7, PSTPIP1, FCHo2, FCHo1 and FBP17 (Figure 1a) showed that positively charged membrane-binding residues found in this region of F-BAR domains are present in CagA (Figure 1a, Table 1). Furthermore, inspection of the alignment of CagA amino acid sequences from 44 representative *H. pylori* strains over the region 613–641 (strain 26695 numbering, see Additional file 1) showed that all of them are either absolutely conserved or conservatively substituted (K/R) (Figure 1b). CagA residues K617, K621, R624 and R626 (equivalent to lipid-binding residues K27, K30, K33 and R35 of FBP17) were shown to bind PS [13, 16]. The observation that the conserved positively charged residues play a similar role in CagA and F-BAR domains is consistent with the functional commonality between the positively charged clusters in CagA and in F-BAR domains - both serve as a membrane-targeting module.

### Mapping homology regions on three-dimensional (3D) structure of F-BAR domains and CagA

**Figure 2:**
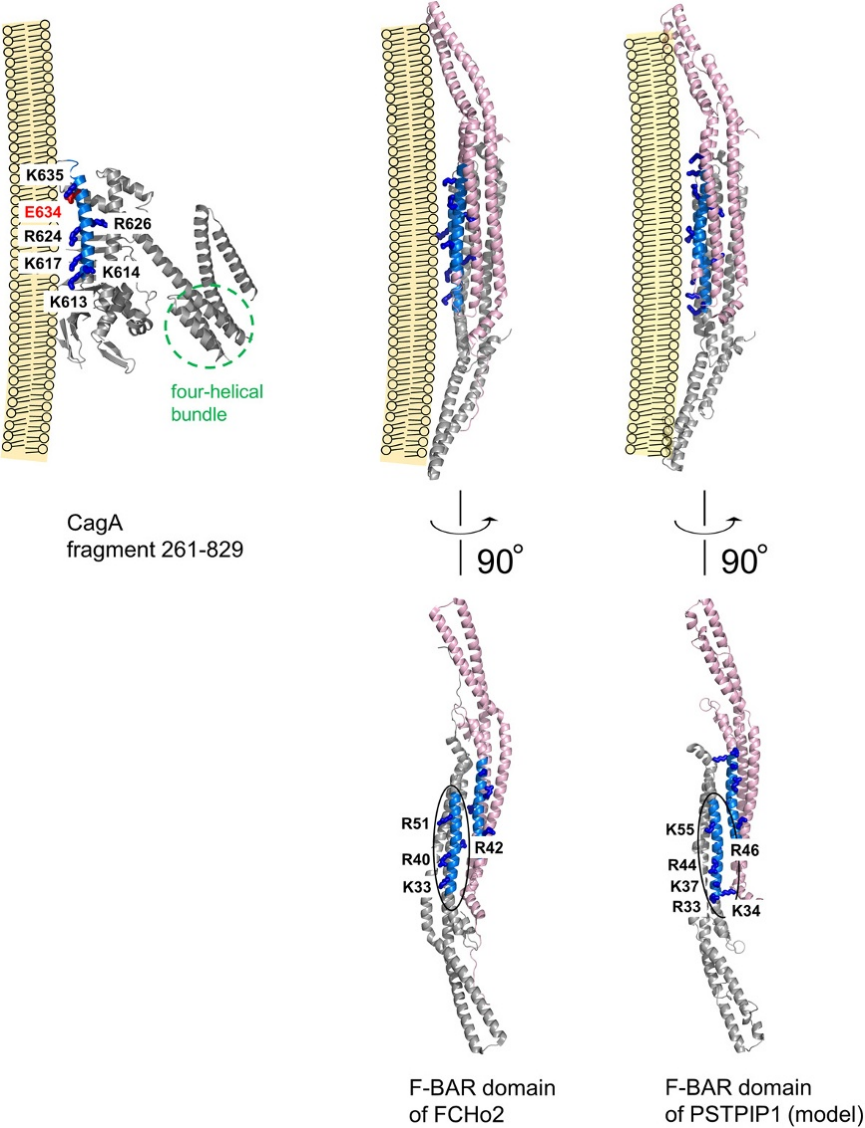
**The membrane-binding residues of the F-BAR domains and the equivalent residues in CagA.** The structures are shown for the F-BAR domains from human FCHo2 (available in the Protein Data Bank (PDB) under code 2v0o [21]), human PSTPIP1 (homology model generated using the above structure 2v0o as a template), and for CagA fragment 261–829 (PDB code 4dvz [16]). The homology region is coloured blue. The side chains of the membrane-binding residues of the F-BAR domains that are conserved in CagA are shown as blue sticks and labelled. The four-helical bundle in CagA that shares structural similarities with proteins that interact with actin-regulatory networks [33] is encircled with a green dashed line. The side chain of E634 in CagA is coloured red to highlight the site with positive selection [34].

Mapping of the membrane-binding residues of the F-BAR domains and the equivalent positively charged residues of CagA on their respective 3D structures [16, 21] (Figure 2) reveals that although the homologous regions of CagA and F-BAR domains share similar (predominantly α-helical) secondary structure, their positions within the overall proteins' folds are distinctly different. The lipid-binding residues of the F-BAR domains reside on a three-helix coiled-coil structure. The region of local homology with CagA (blue in Figure 2) is formed by the residues of helix α2b. In contrast, the equivalent residues in CagA are located on a helix (α18) that forms part of the Domain II [16], comprising an extended single-layer β-sheet and two helical subdomains. Furthermore, unlike CagA which is monomeric *in vitro* [16, 32], F-BAR domains are obligate dimers (Figure 2), and the shape of their dimeric structure is important for their function in membrane recognition and bending [23–29]. As illustrated in Figure 2, the region in F-BAR domains that shows homology with CagA binds membranes as a dimer. Given these overall topological differences, are there common local structural features in these regions that explain why both function as a membrane tether? Inspection of the structures and the local sequence alignment (Figure 1) shows that these helices possess an amphipathic nature with the hydrophobic face hidden in the protein core, and the hydrophilic face exposed to the solvent. The positively charged membrane-binding residues that are conserved between F-BAR domains and CagA align on the hydrophilic side, facing the negatively charged phosphate groups of the lipid membrane (Figure 2), thus promoting favorable interaction between the protein and phospholipid bilayer.

## Discussion

Membrane tethering of the bacterial-borne effector protein CagA plays an essential role in its pathogenic activity. Upon T4SS-mediated translocation into the host cell, CagA is localized to the inner surface of the cell membrane and phosphorylated by the membrane-associated Src kinases. The phosphorylated CagA recruits SHP-2 to the plasma membrane, where it activates SHP-2 phosphatase activity. Activated SHP-2 then dephosphorylates substrates that are also located in close proximity to the membrane and thereby generates signals that lead to morphological changes of the gastric cell. In addition to its role in the intracellular function of CagA, interaction with phospholipids, and specifically PS in the outer membrane of the host cell, is important for translocation of CagA across the host cell membrane [13], the mechanism of which remains to be elucidated.

The interaction interface between CagA and the phospholipid membrane is known to involve several separate sites in the protein. The positively charged helix α18 (residues 610–639), harboring a surface-exposed cluster of conserved lysine/arginine residues at positions 613, 614, 617, 621, 624, 626, 631, 635 and 636, is believed to tether CagA to the negatively charged phosphate groups of the lipid membrane *via* electrostatic interactions [13, 16]. Although the first 200 amino acids of CagA have been shown to be sufficient for membrane tethering [35], regions 200–800 and 800–1216 were subsequently shown to also be important for membrane binding [36], leading to a hypothesis that two separate domains within the C-terminal region, spanning residues 200–800 and 800–1216, interact *in trans* to mediate interactions with the host cell membrane.

The analysis presented here reveals a previously unsuspected similarity between the membrane-tethering helices of CagA and eukaryotic F-BAR domains, thus providing a new insight into the molecular mechanisms underpinning interaction of CagA with lipid membranes of human epithelial cells. The discovery that, despite the low overall sequence identity and distinctly different protein folds, many positively charged residues found on the lipid binding face of the F-BAR domains are conserved in CagA and represent residues involved in CagA binding to lipids suggests that the effector protein CagA acquired a similar function through convergent evolution. In line with this finding, CagA and F-BAR domains have similar lipid specificity profiles. All BAR superfamily members, including F-BAR domains, bind to the plasma membrane through electrostatic interactions with negatively charged phospholipids, showing high affinity to PS and phosphoinositides such as phosphatidylinositol (PI) 4,5-bisphosphate (PI(4,5)P2) and PI 3,4,5-triphosphate (PI(3,4,5)P3) [21, 27, 30, 31]. Similarly, *H. pylori* CagA strongly binds PS and phosphoinositides, including PI 3-phosphate (PI3P), PI4P, PI5P, PI(3,4)P2, PI(3,5)P2 and PI(4,5)P2 [16]. Interaction of CagA with the host membrane PS, which is aberrantly externalized at the site of *H. pylori* attachment, plays an essential role in the translocation of CagA across the host cell membrane and subsequent CagA localization to its inner leaf, which is central to the pathophysiological activity of this protein [13]. The CagA region of homology to F-BAR domains (amino acids 613–641) resides entirely within the boundaries of the PS-binding domain mapped by previous studies [14, 16]. Many of the positively charged residues important for the PS binding by CagA (K613, K614, K617, K621, R624, R626, K631, K635 and K636) are conserved in F-BAR domains, where their role is also to specifically recognise PS and phosphoinositides. This supports the notion of a functional commonality between these positively charged clusters in CagA and in F-BAR domains which convergently evolved as eukaryotic membrane-targeting modules.

This conclusion is in line with a recent study of the evolution of the *cagA* gene by Furuta *et* al. [34] which revealed that region 613–641 contains a site (amino acid 634) that has undergone positive selection. The side chain of the residue at this position points towards the putative interface with the negatively charged membrane surface (Figure 2). The local sequence alignment (Figure 1) shows that, in contrast to CagA from *H. pylori* strain 26695 where this position is occupied by the acidic (glutamate) residue, many F-BAR domains have a small residue at this site (alanine, glycine, serine), which would be more favourable for the interaction with the negatively charged membrane surface. From this point of view, it is important to note that, as shown by Furuta *et* al. [34], in the course of adaptive evolution, many Eastern (more pathogenic) strains of *H. pylori* have also acquired a small residue (alanine or valine) at this position, whereas a significant proportion of Western (less pathogenic) strains have glutamate. This observation raises an interesting possibility that CagA in more pathogenic strains evolved to bind to membranes with higher affinity. This hypothesis should be tested experimentally in future.

**Figure 3:**
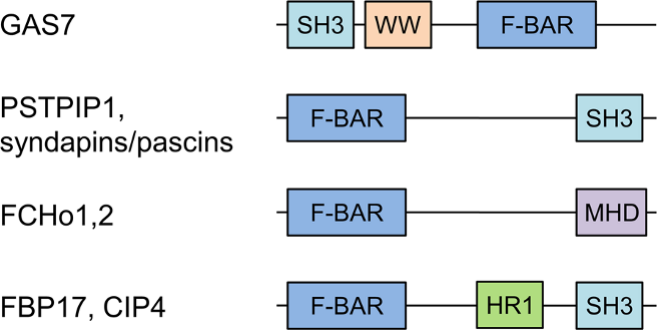
**Domain architecture of the representative F-BAR proteins.** GAS7, growth arrest-specific protein 7; PSTPIP1, proline-serine-threonine phosphatase-interacting protein 1; FCHo1,2, FCH domain only proteins 1 and 2; FBP17, formin-binding protein 17; CIP4, Cdc42 interacting protein 4.

Further functional parallels between the bacterial effector protein CagA and eukaryotic F-BAR domain containing proteins can be drawn when one considers their respective mechanisms of action. Members of the F-BAR domain protein subfamily are typically linked to reorganization of the actin cytoskeleton [21, 27, 31, 37, 38]. In addition to the membrane-binding F-BAR domain, they usually contain other domains (*e. g.* SH3, WW, MHD, HR1 (Figure 3)) that interact with actin-regulatory networks. These proteins are often found at endocytic sites where they mediate interplay between membrane dynamics and cytoskeletal components by binding to the neck of the endocytic vesicle *via* the F-BAR domain and recruiting *via* the other domain, factors that initiate actin polymerisation for vesicle budding. One of the well-characterized biological functions of *H. pylori* CagA is the drastic change of the morphology of gastric cells (elongation) caused by CagA-mediated deregulation of the actin cytoskeleton [39]. Thus, like eukaryotic F-BAR domain proteins, *H. pylori* CagA function appears to be linked to actin dynamics. Similar to the F-BAR proteins, CagA contains a subdomain (four-helical bundle located at the end of helix α19 and comprising helices α19-α22, (Figure 2)) that shares structural similarities with proteins that interact with actin-regulatory networks, such as the F-actin binding domain of the Bcr-Abl tyrosine kinase, α-catenin and vinculin [33]. The uncovered similarities between the bacterial effector protein CagA and eukaryotic F-BAR proteins that are implicated in endocytosis suggests convergent evolution of CagA towards a similar function and raises the question of whether secreted CagA can facilitate its own uptake into human epithelial cells *via* an endocytosis-like process.

## Electronic supplementary material

Additional file 1: **Alignment of CagA amino acid sequences from 44 different** ***H. pylori*** **strains over the region 613-641 (strain 26695 numbering).** (PDF 59 KB)

## References

[1] Covacci A, Telford JL, Del Giudice G, Parsonnet J, Rappuoli R (1999). *Helicobacter pylori* virulence and genetic geography. Science.

[2] Marshall BJ, Warren JR (1984). Unidentified curved bacilli in the stomach of patients with gastritis and peptic ulceration. Lancet.

[3] Peek RM, Blaser MJ (2002). *Helicobacter pylori* and gastrointestinal tractadenocarcinomas. Nat Rev Cancer.

[4] Uemura N, Okamoto S, Yamamoto S, Matsumura N, Yamaguchi S, Yamakido M, Taniyama K, Sasaki N, Schlemper RJ (2001). *Helicobacter pylori* infection and the development of gastric cancer. N Engl J Med.

[5] Backert S, Tegtmeyer N, Selbach M (2010). The versatility of *Helicobacter pylori* CagA effector protein functions: the master key hypothesis. Helicobacter.

[6] Higashi H, Tsutsumi R, Muto S, Sugiyama T, Azuma T, Asaka M, Hatakeyama M (2002). SHP-2 tyrosine phosphatase as an intracellular target of *Helicobacter pylori* CagA protein. Science.

[7] Jones KR, Whitmire JM, Merrell DS (2010). A tale of two toxins: *Helicobacter pylori* CagA and VacA modulate host pathways that impact disease. Front Microbiol.

[8] Murata-Kamiya N (2011). Pathophysiological functions of the CagA oncoprotein during infection by *Helicobacter pylori*. Microbes Infect.

[9] Selbach M, Moese S, Hauck CR, Meyer TF, Backert S (2002). Src is the kinase of the *Helicobacter pylori* CagA protein *in vitro* and *in vivo*. J Biol Chem.

[10] Stein M, Bagnoli F, Halenbeck R, Rappuoli R, Fantl WJ, Covacci A (2002). c-Src/Lyn kinases activate *Helicobacter pylori* CagA through tyrosine phosphorylation of the EPIYA motifs. Mol Microbiol.

[11] Censini S, Lange C, Xiang Z, Crabtree JE, Ghiara P, Borodovsky M, Rappuoli R, Covacci A (1996). *cag*, a pathogenicity island of *Helicobacter pylori*, encodes type I-specific and disease-associated virulence factors. Proc Natl Acad Sci USA.

[12] Odenbreit S (2000). Translocation of *Helicobacter pylori* CagA into gastric epithelial cells by type IV secretion. Science.

[13] Murata-Kamiya N, Kikuchi K, Hayashi T, Higashi H, Hatakeyama M (2010). *Helicobacter pylori* exploits host membrane phosphatidylserine for delivery, localization, and pathophysiological action of the CagA oncoprotein. Cell Host Microbe.

[14] Bagnoli F, Buti L, Tompkins L, Covacci A, Amieva MR (2005). *Helicobacter pylori* CagA induces a transition from polarized to invasive phenotypes in MDCK cells. Proc Natl Acad Sci USA.

[15] Saadat I, Higashi H, Obuse C, Umeda M, Murata-Kamiya N, Saito Y, Lu H, Ohnishi N, Azuma T, Suzuki A, Ohno S, Hatakeyama M (2007). *Helicobacter pylori* CagA targets PAR1/MARK kinase to disrupt epithelial cell polarity. Nature.

[16] Hayashi T, Senda M, Morohashi H, Higashi H, Horio M, Kashiba Y, Nagase L, Sasaya D, Shimizu T, Venugopalan N, Kumeta H, Noda NN, Inagaki F, Senda T, Hatakeyama M (2012). Tertiary structure-function analysis reveals the pathogenic signaling potentiation mechanism of *Helicobacter pylori* oncogenic effector CagA. Cell Host Microbe.

[17] Geer LY, Domrachev M, Lipman DJ, Bryant SH (2002). CDART: protein homology by domain architecture. Genome Res.

[18] Cole C, Barber JD, Barton GJ (2008). The Jpred 3 secondary structure prediction server. Nucleic Acids Res.

[19] Sali A, Blundell TL (1993). Comparative protein modelling by satisfaction of spatial restraints. J Mol Biol.

[20] Fiser A, Do RK, Sali A (2000). Modeling of loops in protein structures. Protein Sci.

[21] Henne WM, Kent HM, Ford MG, Hegde BG, Daumke O, Butler PJ, Mittal R, Langen R, Evans PR, McMahon HT (2007). Structure and analysis of FCHo2 F-BAR domain: A dimerizing and membrane recruitment module that effects membrane curvature. Structure.

[22] DeLano WL (2003). The PyMOL Molecular Graphics System. Version 0.90.

[23] Shimada A, Niwa H, Tsujita K, Suetsugu S, Nitta K, Hanawa-Suetsugu K, Akasaka R, Nishino Y, Toyama M, Chen L, Liu ZJ, Wang BC, Yamamoto M, Terada T, Miyazawa A, Tanaka A, Sugano S, Shirouzu M, Nagayama K, Takenawa T, Yokoyama S (2007). Curved EFC/F-BAR-domain dimers are joined end to end into a filament for membrane invagination in endocytosis. Cell.

[24] Rao Y, Ma Q, Vahedi-Faridi A, Sundborger A, Pechstein A, Puchkov D, Luo L, Shupliakov O, Saenger W, Haucke V (2010). Molecular basis for SH3-domain-mediated regulation of F-BAR-mediated membrane deformation. Proc Natl Acad Sci USA.

[25] Frost A, De Camilli P, Unger VM (2007). F-BAR proteins join the BAR family fold. Structure.

[26] Frost A, Perera R, Roux A, Spasov K, Destaing O, Egelman EH, De Camilli P, Unger VM (2008). Structural basis of membrane invagination by F-BAR domains. Cell.

[27] Itoh T, Erdmann KS, Roux A, Habermann B, Werner H, De Camilli P (2005). Dynamin and the actin cytoskeleton cooperatively regulate plasma membrane invagination by BAR and F-BAR proteins. Dev Cell.

[28] McPherson VA, Everingham S, Karisch R, Smith JA, Udell CM, Zheng J, Jia Z, Craig AW (2009). Contributions of F-BAR and SH2 domains of Fes protein tyrosine kinase for coupling to the FcϵRI pathway in mast cells. Mol Cell Biol.

[29] Reider A, Barker SL, Mishra SK, Im YJ, Maldonado-Báez L, Hurley JH, Traub LM, Wendland B (2009). Syp1 is a conserved endocytic adaptor that contains domains involved in cargo selection and membrane tubulation. EMBO J.

[30] Edeling MA, Sanker S, Shima T, Umasankar PK, Höning S, Kim HY, Davidson LA, Watkins SC, Tsang M, Owen DJ, Traub LM (2009). Structural requirements for PACSIN/syndapin operation during zebrafish embryonic notochord development. PLoS ONE.

[31] Tsujita K, Suetsugu S, Sasaki N, Furutani M, Oikawa T, Takenawa T (2006). Coordination between actin cytoskeleton and membrane deformation by a novel membrane tubulation domain of PCH proteins is involved in endocytosis. J Cell Biol.

[32] Woon AP, Tohidpour A, Alonso A, Saijo-Hamano Y, Kwok T, Roujeinikova A (2013). Conformational analysis of isolated domains of *Helicobacter pylori* CagA. PLoS ONE.

[33] Kaplan-Türköz B, Jiménez-Soto LF, Dian C, Ertl C, Remaut H, Louche A, Tosi T, Haas R, Terradot L (2012). Structural insights into *Helicobacter pylori* oncoprotein CagA interaction with β1 integrin. Proc Natl Acad Sci U S A.

[34] Furuta Y, Yahara K, Hatakeyama M, Kobayashi I (2011). Evolution of *cagA* oncogene of *Helicobacter pylori* through recombination. PLoS ONE.

[35] Pelz C, Steininger S, Weiss C, Coscia F, Vogelmann R (2011). A novel inhibitory domain of *Helicobacter pylori* protein CagA reduces CagA effects on host cell biology. J Biol Chem.

[36] Steininger S, Pelz C, Vogelmann R (2011). Purpose of recently detected inhibitory domain of the *Helicobacter pylori* protein CagA. Gut Microbes.

[37] She BR, Liou GG, Lin-Chao S (2002). Association of the growth-arrest-specific protein Gas7 with F-actin induces reorganization of microfilaments and promotes membrane outgrowth. Exp Cell Res.

[38] You JJ, Lin-Chao S (2010). Gas7 functions with N-WASP to regulate the enurite outgrowth of hippocampal neurons. J Biol Chem.

[39] Wessler S, Gimona M, Rieder G (2011). Regulation of the actin cytoskeleton in *Helicobacter pylori*-induced migration and invasive growth of gastric epithelial cells. Commun Signal.

