# Supplementary material for: Phospholipid binding residues of eukaryotic membrane-remodelling F-BAR domain proteins are conserved in Helicobacter pylori CagA
Source: BMC Res Notes. 2014 Aug 13;7:525. doi: 10.1186/1756-0500-7-525 (PMC4141123; doi:10.1186/1756-0500-7-525)
Supplement: Supplementary file 1 — Additional file 1: Alignment of CagA amino acid sequences from 44 different H. pylori strains over the region 613-641 (strain 26695 numbering). (PDF 59 KB) [file 13104_2014_3052_MOESM1_ESM.pdf]

Alignment of CagA amino acid sequences from 44 different *H. pylori* strains over the region 613-641 (strain 26695 numbering)

Genbank ID				Strain
AAD07614	613	KKAQKDLEKSLRKREHLEKEVEKKLESKS	641	26695
ACI27269	608	KQAQKDLEKSLKKRERLEKEVAKKLESKS	636	G27
AAD06073	612	KQAQKDLEKSLKKRERLEKDVAKNLESKS	640	J99
ACJ07709	608	KKAQKDLEKSLKKREHLEKEVAKNLESKS	636	P12
CBI66298	612	KKAQKNLEKSLRKREHLEKEVVKKLENRN	640	B8
ADN79679	608	KRAQKDLEKSLKKREHLEKDVAKNLESKS	636	908
BAD51763	613	KKAQKDLEKSLRKREHLEKEVAKKLESRN	641	OK197
BAD51766	617	KKAQKDLEKSLRKREHLEKEVTKKLERKS	645	OK210
BAD51757	611	KKAQKDLEKSLRKREHLEKEVTKKLERKS	639	OK168
CBV36429	613	KKAQKDLEKSLRKREHLEKEVAKKLESRN	641	M49
BAD13990	611	KKAQKDLEKSLRKREHLEKEVTKKLERKS	639	OK107
BAD13908	608	KKAQKDLEKSLRKREHLEKEVEKKLESKS	636	F79
BAD14045	608	KRTQKDLEKSLKKREHLEKGVVKNLESKS	636	OK112
CBV35907	617	KKAQKDLEKSLRKREHLEKEVAKKLESKS	645	101UK
CBV36031	608	KRAQKDLEKSLKKRERLEKDVAKNLESKS	636	D3a
CBV36400	608	KRAQKDLEKSLKKREHLEKDVAKNLESKS	636	LSU2003-1
CBV36458	608	KKAQKDLEKSLRKREHLEKEVEKKLESKS	636	MOR3457
CBV35937	608	KKAQKDLEKSLRKREHLEKEVEKKLESKS	636	BASQ8846
CBV36254	612	KKAQKDLEKSLRKREHLEKEVTKKMESKS	640	KAZ3173
CBV36546	607	KKAQKDLEKSLRKREHLEKEVVKKLERKS	635	PAL3414
CBV35967	608	KKAQKDLEKSLRKREHLEKEVEKKLESKS	636	CC33C
CBV36090	607	KKAQKDLEKSLKKREHLEKEVAKNLESKS	635	FIN9624
CBV36119	608	KKAQKDLEKSLRKREHLEKDVAKNLESKS	636	H1419
CBV36664	608	KQAQKDLEKSLKKRERLEKDVAKNLESKS	636	SU2
CBV36516	608	KKAQKDLEKSLRKREHLEKEVEKKLESKS	636	NQ367
BAD51746	613	KKAQKDLEKSLRKREHLEKEVAKKLESRN	641	F75
BAD51745	607	KKAQKDLEKSLRKREHLEKEVAKKLESRN	635	F57
BAJ58105	609	KKAQKDLEKSLRKREHLEKEVAKKLESRN	637	F32
BAJ56876	613	KKAQKDLEKSLRKREHLEKEVAKKLESRN	641	F30
BAJ55408	609	KKAQKDLEKSLRKREHLEKEVAKKLESRN	637	F16
BAD51748	617	KKAQKDLEKSLRKREHLEKEVTKKLESRN	645	OK113
BAD51743	613	KKAQKDLEKSLRKREHLEKEVAKKLESRN	641	F24
BAD51765	613	KKAQKDLEKSLRKREYLEKEVAKKLESRN	641	OK206
BAD51742	609	KKAQKDLEKSLRKREHLEKEVAKKLESRN	637	F23
BAD51767	609	KKAQKDLEKSLRKREHLEKEVAKKLESRN	637	OK212
ADU41149	613	KKAQKDLEKSLRKREHLEKEVAKKLESRN	641	35A
BAD14072	609	KKAQKDLEKSLRKREHLEKEVAKKLESRN	637	OK129
BAD14018	613	KKAQKDLEKSLRKREHLEKEVAKKLESRN	641	OK109
BAD13853	613	KKAQKDLEKSLRKRELLEKEVAKKLESRN	641	F28
BAD13826	613	KKAQKDLEKSLRKREHLEKEVAKKLESRN	641	F17
BAD13963	609	KKAQKDLEKSLRKREHLEKEVAKKLESRN	637	OK101
CBV36633	613	KKAQKDLEKSLRKREHLEKEVAKKLESRN	641	RE12001
CBV36487	613	KKAQKDLEKSLRKREHLEKEVAKKLENRN	641	N2
CBV36061	613	KKAQKDLEKSLRKREHLEKEVAKKLESRN	641	DU15
